# Fish communities in coastal freshwater ecosystems: the role of the physical and chemical setting

**DOI:** 10.1186/1472-6785-8-23

**Published:** 2008-12-29

**Authors:** Kristin K Arend, Mark B Bain

**Affiliations:** 1 Department of Natural Resources, Cornell University, Fernow Hall, Ithaca, NY, 14853, USA; 2 Department of Forestry and Natural Resources, Purdue University, West Lafayette, IN, 47907, USA

## Abstract

**Background:**

We explored how embayment watershed inputs, morphometry, and hydrology influence fish community structure among eight embayments located along the southeastern shoreline of Lake Ontario, New York, USA. Embayments differed in surface area and depth, varied in their connections to Lake Ontario and their watersheds, and drained watersheds representing a gradient of agricultural to forested land use.

**Results:**

We related various physicochemical factors, including total phosphorus load, embayment area, and submerged vegetation, to differences in fish species diversity and community relative abundance, biomass, and size structure both among and within embayments. Yellow perch (*Perca flavescens*) and centrarchids numerically dominated most embayment fish communities. Biomass was dominated by piscivorous fishes including brown bullhead (*Ameiurus nebulosus*), bowfin (*Amia calva*), and northern pike (*Esox lucius*). Phosphorus loading influenced relative biomass, but not species diversity or relative abundance. Fish relative abundance differed among embayments; within embayments, fish abundance at individual sampling stations increased significantly with submerged vegetative cover. Relative biomass differed among embayments and was positively related to total phophorus loading and embayment area. Fish community size structure, based on size spectra analysis, differed among embayments, with the frequency of smaller-bodied fishes positively related to percent vegetation.

**Conclusion:**

The importance of total phosphorus loading and vegetation in structuring fish communities has implications for anthropogenic impacts to embayment fish communities through activities such as farming and residential development, reduction of cultural eutrophication, and shoreline development and maintenance.

## Background

Physicochemical features at multiple spatial scales (e.g., watershed, embayment, and habitat) can be important for fish community structure [1-3]. Variability in nutrient inputs, hydrology, and morphometry among and within aquatic ecosystems can shape fish communities [1,4-6]. In turn, fish community structure influences ecosystem function, such as energy transfer and nutrient cycling [7-9] via trophic interactions and, in some cases, habitat modification [10,11]. Consequently, fish communities are important indicators of and interactors in aquatic ecosystems.

We explored how physicochemical features shaped fish communities in Lake Ontario embayments. Great Lakes embayments are relatively shallow, inshore ecosystems located between the shorelines of the lakes and their watersheds. Embayments vary considerably in nutrient loading, hydrology, and morphometry. Additionally, embayments serve as conduits of nutrients and other materials from their watersheds [12,13], support high fish species diversity [12], provide spawning and nursery habitats for both nearshore and offshore Great Lakes fishes [14,3,15], and are concentrated areas of human activities [16]. These characteristics make embayments ideal systems with which to address physicochemical effects on fish community structure in the context of ecosystem function.

Great Lakes embayments range in hydrogeomorphic type, including flooded river mouths, coastal wetlands, and large, deep enclosed bays [17,18,15]. Embayments are connected to their watersheds by tributary inflow, surface runoff, and/or groundwater flow. While some embayments lack direct, surface water connections to the main lake, most embayments have either man-made or natural connections that can be permanent, seasonal, or ephemeral [19]. This combination of morphometric and hydrologic variability results in physicochemical habitat conditions that differ both among and within embayments [16,20]. For example, morphometry and water inflow from tributaries and the lake (via seiches) interact to influence water chemistry, submerged aquatic vegetation, and dissolved oxygen and temperature profiles [20].

We posed the question: how do watershed inputs, hydrology, and embayment morphometry affect fish community structure in eight embayments located along the southeastern coast of Lake Ontario? We expected that variation in these physicochemical factors across spatial scales would influence multiple metrics of fish community structure, including diversity, relative abundance, biomass, and size structure. At the watershed scale, watershed size, discharge and land use affect productivity, which in turn, can influence fish community structure and dynamics. We hypothesized that high nutrient inputs to embayments, from either high watershed flows (i.e., short water residence time) or high nutrient concentrations due to land use, would positively affect fish abundance and biomass [21,22,1] and negatively affect species diversity through loss of intolerant species [4,23]. At the system (i.e., embayment) scale, greater surface area with a more complex depth profile can increase habitat and resource heterogeneity, which positively impact fish abundance, biomass, and diversity [24,6].

Within systems, availability of vegetated, littoral habitat also affects fish communities by increasing habitat and resource heterogeneity [14,25,26]. As such, we predicted that embayments with higher habitat heterogeneity (e.g., large surface area and/or abundant, vegetated littoral habitat) would support more diverse and abundant fish communities than small or more homogeneous embayments. Morphometry also impacts fish community size structure [1,6]. We hypothesized that a higher proportion of small-bodied than large-bodied fishes would occur in shallow embayments dominated by vegetated habitat [1]. In contrast, large embayments having deep, open habitat would provide support for large-bodied fishes [6], resulting in a low proportion of small-bodied fishes due to predation [10,27].

In this paper, we used hierarchical mixed modelling to relate differences among embayment fish communities to abiotic and biotic factors at the watershed through sampling station scales. The response variables we considered were fish community species diversity, relative biomass and abundance, and size structure. Predictor variables included total phosphorus load, embayment area, sampling station depth, percent aquatic vegetation, and percent littoral habitat and, for size structure only, piscivore relative biomass.

## Methods

### Study site hydrogeomorphic classification

**Figure 1:**
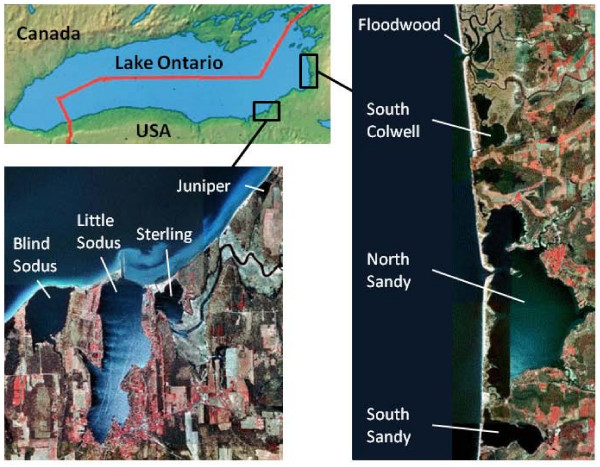
**Embayment locations**. Location map of the eight Lake Ontario study embayments.

Study embayments were located in two clusters along the southeastern shoreline of Lake Ontario, New York, USA (Figure 1) and varied in several watershed and embayment characteristics (Additional file 1). For purposes of this research, we classified embayments into general hydrogeomorphic types, based on Keough et al. [17]: (1) drowned-river mouth embayments (Sterling, Floodwood); (2) pelagic-protected embayments (Blind Sodus, Little Sodus, South Sandy); and (3) littoral-protected embayments (Juniper, North Sandy, and South Colwell). Drowned-river mouth embayments receive high watershed inputs, have short water residence times, and have a surface water connection with Lake Ontario [17]. Protected embayments have longer water residence times than drowned-river mouths, are separated from Lake Ontario by a sand barrier, and vary in their hydrologic connections to their watershed and Lake Ontario [17]. We defined pelagic-protected embayments as having depths that exceed euphotic zone depth estimates for at least 10% of their area. Littoral-protected embayment depths do not exceed euphotic zone depth estimates.

### Embayment-scale characteristics

#### Morphometry

Morphometric measurements included watershed area, embayment area, maximum depth, and percent littoral habitat. Watershed areas were calculated from digital elevation maps using ESRI ArcHydro tools [28]. Annual embayment area and maximum depth were calculated from bathymetric maps and annual averages of Lake Ontario water level for each year of the study (NOAA, Oswego, NY). Bathymetric maps were generated from elevations that were calculated using depth measurements taken in the field and, for reference over time, the 1985 Lake Ontario water level of 74.67 meters (m; NOAA, Oswego, NY).

Percent littoral habitat was calculated from bathymetric maps based on embayment-specific estimates of euphotic zone depth, i.e., the depth at which 1% incident light intensity occurs. Mean Secchi disk depth (m) for each embayment was calculated using data collected weekly from May through mid-October in 2001 and 2002, and biweekly from June through mid-October 2003 at centrally located stations in each embayment. Euphotic zone depth was estimated as 2.7 * mean Secchi disk depth [29].

Depths less than or equal to the euphotic zone depth in each embayment were defined as littoral; depths greater than the euphotic zone depth were defined as pelagic. Littoral and pelagic areas (km^2^) in each embayment were estimated from bathymetric maps using Arcview GIS 3.x [30]. Euphotic zone depth estimates for Juniper and South Colwell exceeded maximum depths in these embayments; therefore, 100% of the habitat was considered littoral, which matched field observations.

#### Water residence time and water chemistry

Annual water residence time and water chemistry were estimated from monthly sampling data provided by X. Chen (Syracuse University, unpublished data). Embayment water residence time was estimated from of the relative contributions of the watershed, Lake Ontario, and direct precipitation to each embayment, as determined by fluoride mass balance calculations (X. Chen, Syracuse University, personal communication). Total phosphorus loading to the embayments was calculated by multiplying stream discharge into the embayments by the input phosphorus concentration (X. Chen, Syracuse University, personal communication).

### Station-scale characteristics

On one or two consecutive dates in July 2001, 2002, and 2003, each embayment was sampled at between three and eight stations, based on embayment size. Embayment bathymetric maps superimposed with a 30 × 30 m grid were divided into three to eight strata. Grid intersection points at which the water column depth was less than or equal to four m were assigned numbers. Each year, sample stations were determined by randomly selecting one grid intersection point from each stratum. This design ensured that stations sampled were distributed throughout the embayments. Embayment sample stations were located using a Global Positioning System (GPS) set to the Universal Transverse Mercator coordinate system, and marked with a buoy (henceforth center).

Habitat data were collected at all stations within each embayment between 0800 and 1800 hours. Mean bottom depth was calculated from bottom depth measurements taken to the nearest 0.1 m at four locations 30 m out in each direction from the center. Visual estimates of the percent of sediment surface supporting submerged aquatic vegetation growth were made at one second time intervals while driving along a circular path approximately 30 m radius from the center. Mean cover for each station was calculated from the estimates. At the center, surface temperature was measured to the nearest 0.1°C with a standard thermometer and Secchi depth was measured to the nearest 0.1 m following standard methods.

### Fish sampling

Fish sampling coincided with sampling for station-level characteristics. At each station, fish were collected using a 4.6 m boat equipped with a Smith-Root Type VI-A electrofishing unit and a 5000 watt generator. The transformer was set at 120 pulses per second direct current electricity, with either 125 or 250 volts and pulse width varying between 7–9 milliseconds. Several factors that can influence fish captured using electrofishing include fish size, water clarity, water depth, and macrophyte density. We tried to minimize unequal bias in catch among embayments by focusing our sampling to concentrated areas at depths less than or equal to four m. Fish were collected along 15 minute (min) inward spirals starting at approximately a 30 m radius from the center of each station. In 2001 and 2002, fish were sampled between 0800 and 1800 hours; in 2003, fish were sampled between 1300 and 2300 hours. Fish were identified to species and total length (TL) was measured to the nearest 1.0 millimeter (mm). In 2002 and 2003, the wet weight in grams (g) of all fish was measured to the nearest 0.1 or 0.5 g. Fish were not weighed in 2001.

As all of our sampling stations were in less than four m deep water, discussions in this paper focus on littoral fish assemblages. However, gill net sampling conducted in littoral and pelagic habitats in Blind Sodus, Little Sodus, South Sandy, and North Sandy during late June – early July, 2002, yielded relatively few fish in pelagic habitat (89 fish, 3293 min total effort) compared with littoral habitat (215 fish, 3223 min total effort). Of fish captured in pelagic habitat, 18% were alewife (*Alosa pseudoharengus*) and 76% were yellow perch (*Perca flavescens*). Therefore, with the exception of alewife, the majority of fishes occurred at depths less than four m, and a unique offshore community was not detected. These data suggest that fishes captured at depths less than four m represented the majority of fishes that occupied the embayments during May through August.

### Data Analysis

Fish data were analyzed either at the community or taxonomic levels. Taxonomic analyses concentrated on nine focal species. Eight of these occurred in relatively high numbers across all embayments and represented a range of trophic positions and feeding habits (e.g., planktivore, invertivore, piscivore): brown bullhead (*Ameiurus nebulosus*), bowfin (*Amia calva*), bluegill (*Lepomis macrochirus*), golden shiner (*Notemigonus crysoleucas*), largemouth bass (*Micropterus salmoides*), northern pike *(Esox lucius*), pumpkinseed (*Lepomis gibbosus*), and yellow perch. Walleye (*Sander vitreus*) was identified as a ninth focal species due to high densities in South Sandy.

#### Fish community structure

Fish species diversity was calculated by aggregating the number of fishes in each species collected across years. Diversity was estimated using Simpson's index (D^-1^), because of its low sensitivity to sample size [31], which varied across embayments. We used ordinary least squares (OLS) linear regression to determine if species diversity was related to total phosphorus loading or to embayment area, which were log-transformed (ln) to reduce heterogeneity of variances.

Weights for all fish captured in 2001 were estimated using species-specific length-weight regressions either generated from data we collected in 2002 and 2003 or reported in the literature. We generated length-weight regressions either for individual embayments or for all embayments pooled, depending on the number of individuals per species captured within and across embayments. Annual catch per unit effort (number·min^-1^; CPUE) and biomass per unit effort (g·min^-1^; BPUE) were estimated for each species in each embayment. Within an embayment-year combination, CPUE and BPUE for each taxonomic group and all fish combined were calculated as the total number and biomass, respectively, of individuals caught in that group divided by total embayment sampling effort that year (min; summed across all stations).

Normalized size spectra (NSS) provide a quantitative way to evaluate the distribution of biomass within each embayment's fish community. The method identifies the size class that supports maximum biomass by sorting organisms (i.e., fish) into size classes and plotting total biomass in each size class versus size class. NSS were created for each embayment-year by transforming all fish weights by log base 2 [32]. The sum of transformed weights in each size class was plotted against the transformed weight of the heaviest fish actually recorded in that size class (sensu [33]). The maximum possible weight in a size class (e.g., 1-0.01 = 0.99) was used if no data existed for that size class. We then solved for the points *h *and *k *describing the location (x axis) and height (y axis), respectively, of the parabola vertex as y = *c *+ *b*·x + *a*·x^2^, using ordinary least squares regression. Values for the coefficients *h *and *k *were calculated as

$$h=\frac{-b}{2\cdot a}$$

and

*k *= *c *+ *b*·*h *+ *a*·*h*^2^.

Coefficient *h *approximates the weight class at which the majority of the fish community's biomass is concentrated, and henceforth will be referred to as maximum biomass weight class. Coefficient *k *estimates total biomass at maximum biomass weight class and is correlated with total fish community biomass [32]. Correlations between the coefficients and between each coefficient and fish biomass were calculated. Maximum biomass weight class also was used as a response variable in the community response analyses (see below).

#### Fish community response to embayment and habitat characteristics

We conducted mixed model analyses using PROC MIXED in SAS [34] both to identify differences in fish community descriptors (e.g., CPUE) among embayments and to relate descriptors to embayment physicochemical features. We used a mixed model to account for the hierarchical structure of the data (stations within years within embayments) and for the use of both continuous and categorical variables. Sample sizes for each level were three years, eight embayments, and between three to eight stations per embayment-year (Additional file 2). Community descriptors included: (1) CPUE of all fish combined; (2) BPUE of all fish combined; and (3) the maximum biomass weight class (coefficient *h *of the NSS). CPUE and BPUE data for all fish species combined were square-root transformed to meet the assumption of normality; maximum biomass weight classes were normally distributed. Similar analyses at the taxonomic level were not possible due to highly variable catch among stations, with many zero catches.

Embayment was specified as a random effect, because we assumed the study embayments represent Lake Ontario embayments in general [35]. Year was categorized as a fixed effect, because of the unlikelihood that three consecutive years of data represent a random sample of years [35]. We considered physicochemical variables at both the embayment and station scales to include as predictors in our analyses. Predictor variables were selected based on Pearson correlation coefficients. Selected variables included: year, water residence time, total phosphorus load (natural log transformed), embayment area (natural log transformed), station depth (natural log transformed), percent submerged aquatic vegetation (arcsine transformation), and percent littoral habitat. Percent littoral habitat, was converted to a binomial variable as either pelagic-dominated (less than 60% of bottom depth area falling within the euphotic zone) or littoral-dominated (for this study, all had greater than 95% of bottom depth area falling within the euphotic zone). The following factors were not considered due to correlation: embayment: watershed area, annual nitrogen loading (kg·y^-1^), embayment volume, and embayment mean depth. We did not include Secchi depth, because depth readings frequently were limited by bottom depth or dense macrophyte beds. We did not include temperature or dissolved oxygen in the models, because data collection was limited to sampling times and thus the data do not characterize the thermal and oxygen regimes of the embayments. From May through October, 2001 and 2002, mean temperature at depths less than or equal to four m ranged from 19–21°C across embayments (Additional file 1). Dissolved oxygen differed by less than four mg·Liter (L)^-1 ^across embayments, and was always greater than five mg·L^-1 ^at zero to five m depths.

Piscivorous fish BPUE (untransformed) was included as a fixed effect in the model of the maximum biomass weight classes. Piscivore BPUE included American eel (*Anguilla rostrata*), bowfin, chain pickerel (*Esox niger*), grass pickerel (*Esox americanus vermiculatus*), largemouth bass, longnose gar (*Lepisosteus osseus*), northern pike, smallmouth bass (*Micropterus dolomieu*), walleye, and white perch (*Morone americana*). We included all largemouth bass, despite high catches of young-of-year bass, based on prey fish in diets of largemouth bass as small as 37 mm TL and on findings by Olive et al. [27] that piscivory by high densities of small-bodied largemouth bass can structure fish communities.

For the mixed model analyses of CPUE and BPUE, data were classified according to embayment, year, and station. Mixed model analysis of maximum biomass weight classes was conducted at the embayment scale only, with data classified according to embayment and year. Degrees of freedom were adjusted using the Kenward-Rogers method. To ensure that only uncorrelated variables were included in each model, (1) one of two or more correlated variables was selected or (2) correlated variables were tested in separate model runs (e.g., percent littoral habitat and percent submerged aquatic vegetation). We selected those models that provided the best, most parsimonious fit to the data, based on model covariance estimates and number of parameters.

Two models were run for each response variable to test for variance in the responses among embayments. In the first model, embayment was specified as a random effect; in the second, no random effects were specified, to identify the variation explained by embayment. The test statistic was calculated as the difference between the two models' log-likelihood values. It follows a χ^2 ^distribution, and its p-value is determined by dividing the probability of a greater χ^2 ^for one degree of freedom by two [34]. We used analyses of the full mixed models to identify significant fixed effects for each response variable. The percent of the variation between embayments explained by each full mixed model was calculated as the difference in variance due to embayment between models with and without the physicochemical factors as predictor variables, expressed as a fraction of the variance due to embayment in the model without the physicochemical factors as predictor variables. Within embayment variation was calculated similarly, using the unexplained (i.e., residual) variance estimates for each model in place of variance due to embayment.

## Results

### Fish community structure

Across all embayments, we collected a total of 3475 fishes representing 42 different species and 16 families. Species diversity (Simpson's index) ranged from 2.7 – 6.7 among embayments (Additional file 3), but was not related to total phosphorus loading (r^2 ^= 0.41, p = 0.09), embayment area (r^2 ^= 0.01; p = 0.81), or percent littoral area (r^2 ^= 0.01; p = 0.83).

**Figure 2:**
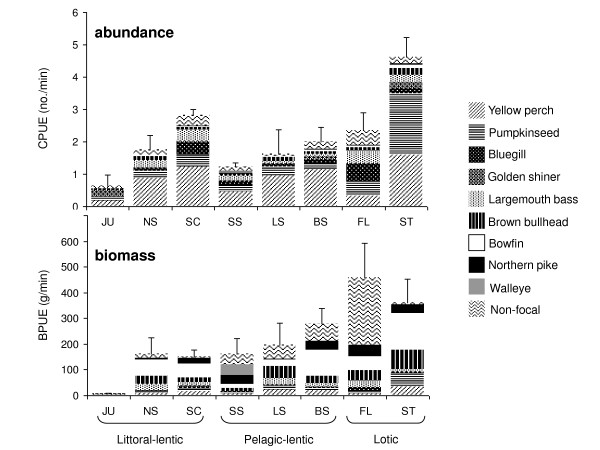
**Fish community relative abundance and biomass**. Relative abundance (total fish catch per unit effort) and biomass (total fish biomass per unit effort) of embayment fish communities. Embayments are shown from left to right in order of increasing phosphorus loading, within each hydrogeomorphic type. Embayment codes are: SC, South Colwell; JU, Juniper; NS, North Sandy; LS, Little Sodus; SS, South Sandy; BS, Blind Sodus; ST, Sterling; and FL, Floodwood. High non-focal species biomass typically is due to the presence of common carp.

Relative abundance (CPUE) and biomass (BPUE) of all fish combined and of individual species varied among embayments and years (Figure 2; Additional file 3). Fish communities were numerically dominated by yellow perch, pumpkinseed, bluegill, and largemouth bass (Figure 2; Additional file 3). With the exception of Floodwood and Juniper, yellow perch constituted between 20–60% (by number) of the fish community. In Floodwood, abundance was more evenly distributed across yellow perch and the centrarchid populations; in Juniper, golden shiner was the numerically dominant species (Figure 2). Large piscivores accounted for the majority of the biomass in all embayments except Juniper, an unconnected embayment where large piscivores were not captured (Figure 2). The most common non-focal species included alewife, common carp (*Cyprinus carpio carpio*), blacknose shiner (*Notropis heterolepis*), common shiner (*Luxilus cornutus*), banded killifish (*Fundulus diaphanus diaphanus *), black crappie (*Pomoxis nigromaculatus*), and smallmouth bass. Large common carp accounted for the high biomass of non-focal species in Blind Sodus, Little Sodus, South Sandy, and Floodwood.

**Figure 3:**
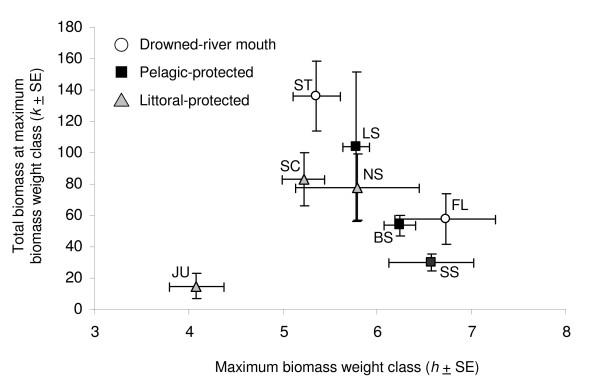
**Embayment fish community biomass at maximum biomass weight class versus maximum biomass weight class**. Total fish community biomass at maximum biomass weight class (*k*) versus maximum fish community biomass weight class (*h*) for drowned-river mouth (open circles), pelagic-protected (black squares), and littoral-protected (grey triangles) embayments. Points represent 3-year means ± standard error (SE). Embayment codes follow Figure 2.

Fish community size structure also varied among embayments and years. Normalized size spectra models captured between 17–71% of the variation in total biomass per weight class for each embayment-year combination. Juniper supported a small-bodied fish community (< 200 mm TL), indicated by a much lower maximum biomass weight class than for the other embayments (Additional file 2; Figure 3). Maximum biomass weight class estimates for all other embayments varied among embayment-year combinations. Maximum biomass weight class in Blind Sodus, South Sandy, and Floodwood occurred at larger weight classes, indicating these fish communities contained a greater proportion of large-bodied fishes than in the other embayments (200–500 mm TL; Additional file 2; Figure 3). Fish biomass was concentrated in medium-sized fish in Little Sodus, Sterling, North Sandy, and South Colwell (Figure 3). Juniper supported a small-bodied fish community (Figure 3). Excluding Juniper, total biomass at the maximum biomass weight class was negatively correlated with maximum biomass weight class (p = 0.002; Figure 3). For all embayments, neither maximum biomass weight class nor total biomass at the maximum biomass weight class was correlated with total fish biomass.

### Fish community response to embayment and habitat characteristics

**Figure 4:**
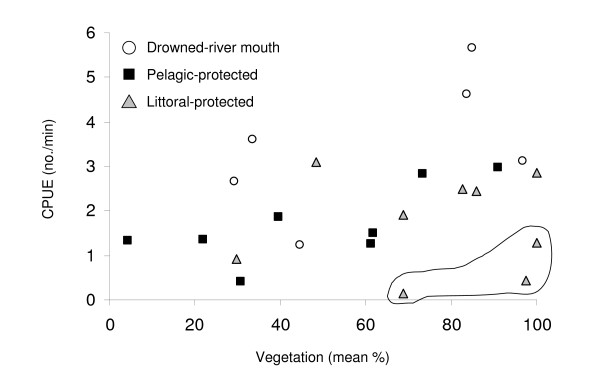
**Fish relative abundance versus mean percent vegetation**. Annual fish relative abundance (total fish catch per unit effort; #·min^-1^) in July, 2001–2003, versus mean percent vegetation for drowned-river mouth (open circles), pelagic-protected (black squares), and littoral-protected (grey triangles) embayments. Circled data outliers are from Juniper, from which relatively few fish were collected.

**Figure 5:**
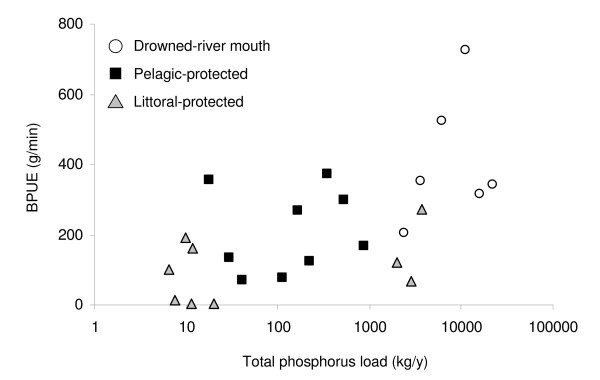
**Fish relative biomass versus total phosphorus loading**. Annual total fish biomass per unit effort (g·min^-1^) in July, 2001–2003, versus total phosphorus loading (kg/y) for drowned-river mouth (open circles), pelagic-protected (black squares), and littoral-protected (grey triangles) embayments.

**Figure 6:**
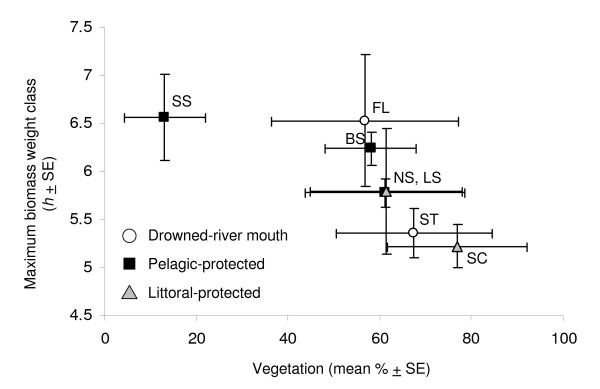
**Maximum biomass weight class versus percent vegetation**. Maximum fish community biomass weight class (h) versus percent vegetation for drowned-river mouth (open circles), pelagic-protected (black squares), and littoral-protected (grey triangles) embayments. Points are 3-year means ± SE. Embayment codes follow Figure 2.

The models that provided the best, most parsimonious fit to the data included total phosphorus load, area, the interaction between total phosphorus load and area, and percent vegetation. Both CPUE and BPUE differed significantly among embayments, as indicated by the likelihood ratio test statistics (p < 0.0025 for both; Table 1). CPUE within embayments was positively related to percent vegetation (p = 0.02; Table 1; Figure 4). BPUE among embayments was positively related to embayment area (p = 0.04) and total phosphorus load (p = 0.02; Table 1; Figure 5) and negatively related to the interaction between area and total phosphorus load (p = 0.03; Table 1). Maximum biomass weight class (the NSS coefficient *h*) also differed among embayments (p = 0.004), and was negatively related to percent vegetation (p = 0.005; Table 1; Figure 6) between embayments.

**Table 1:** Fish community response to physicochemical factors

	**Species diversity**	**CPUE (#·m^2^)**	**BPUE (g·m^2^)**	**Size-structure (*h*)**
Variation by embayment	ns	p < 0.0025	p < 0.0025	p = 0.04
Physicochemical Factor				
TP load (0.88 – 4.34)	ns	ns	28 (0.02)	ns
Area (4.79 – 7.05)	ns	ns	9.6 (0.04)	ns
TPload*Area	ns	ns	-4.3 (0.03)	ns
% vegetation (0 – 90)	ns	0.01 (0.02)	ns	-0.05 (0.005)
Variation model	n/a			
Between		-0.146	0.86	0.83
Within		0.132	0.005	-0.004

"Variation by embayment" indicates if variability for each response among embayments significantly differed from zero. Effects of total phosphorus load (TPload; kg·y^-1^), embayment area (m^2^), and percent vegetation on each characteristic are presented as slope estimates; ns indicates no significant effect of that factor; n/a indicates not applicable. P values for effects are indicated in parentheses under each response column; the range of transformed values for each physicochemical effect are indicated in parentheses to the right of each effect's name. The variation explained by each model ("Variation model") is divided into variation between embayments ("Between") and within embayments ("Within").

## Discussion

Despite similar fish species composition and diversity among embayments, community relative abundance, biomass, and size structure differed among embayments. These differences were correlated with physicochemical attributes at the watershed and embayment scales. Phosphorus loading influenced fish community relative biomass, but not species composition or community relative abundance. Greater fish biomass was supported in large, deep embayments and those receiving high phosphorus loading. Vegetated embayments supported more fish, with biomass concentrated in small-bodied fishes than did less vegetated embayments. Within embayments, stations with greater submerged vegetative cover supported more smaller-bodied fishes. Water residence time did not influence fish community characteristics directly, but could inversely affect phosphorus loading by phosphorus dilution or reduced phosphorus retention at high flows.

Species diversity was not significantly affected by total phosphorus loading, despite a large range in loading. Our sites are located in eastern Lake Ontario, which is less impacted by urban and agricultural activity and receives lower nutrient and sediment inputs from the watershed than western Lake Ontario [18,36]. Among our sites, embayments in the eastern cluster (South Sandy, North Sandy, South Colwell, and Floodwood) are less impacted by land use than those in the western cluster [37], which could explain slightly higher values for species diversity in those embayments. Anthropogenic eutrophication may not be great enough in these systems to alter species composition significantly. Phosphorus loading did affect biomass, however, suggesting that impacts of nutrient enrichment on fish communities can be detected before negative effects such as changes in fish community composition are evident. A slight positive trend in species diversity with phosphorus loading indicates even those embayments with higher loadings were not sufficiently eutrophied to experience loss of intolerant species. We would expect even greater variation among embayments in fish biomass and possibly loss of species diversity at the highest levels of loading, if the entire range of phosphorus loading to Lake Ontario embayments had been included in our study.

As shown in other studies, fish biomass increased with total phosphorus loading (e.g., [21,22,1]) and embayment area (e.g., [24,6]). In contrast, however, phosphorus inputs and area did not influence fish relative abundance. Relative fish biomass may have been more sensitive to phosphorus loading than abundance because biomass more accurately represents the amount of fish tissue that must be supported. The positive effect of embayment area on biomass was reduced as total phosphorus load increased, and vice versa. Differences among hydrogeomorphic types in the relative importance of area and phosphorus may explain this relationship. For example, fish biomass was greatest in the drowned-river mouth embayments (Sterling and Floodwood), which also received the highest nutrient loading but are two of the smaller embayments. Both area and productivity appeared to influence fish biomass in the pelagic-protected embayments (Little Sodus, South Sandy, and Blind Sodus). For example, high phosphorus loading to Blind Sodus resulted in high biomass despite it being the smallest of the three embayments, whereas the large size of Little Sodus resulted in it supporting an intermediate amount of biomass, despite very low phosphorus loading. Neither size nor productivity seems to explain fish biomass in the littoral-protected embayments (Juniper, South Colwell, and North Sandy). Although North Sandy is the largest embayment and receives high phosphorus loading, it supports similar fish biomass to South Colwell, a small embayment with little loading. Therefore, other factors, such as habitat availability within embayments, may be more important in structuring littoral-protected fish communities.

Indeed, aquatic vegetation has been identified as an important factor in structuring fish communities in shallow, littoral-dominated systems [1,26]. Randall et al. [1] found that fish were more numerous and smaller sized in vegetated versus unvegetated littoral habitat in Lake Ontario and Lake Huron bays, but that fish biomass did not differ. Our results complement those findings, even when considering more pelagic-dominated systems. For example, the two drowned-river mouth systems, Sterling and Floodwood, supported similarly high fish biomass; however, numerous, small-bodied fishes dominated the fish community in Sterling (with dense macrophyte beds), whereas fewer but larger-bodied fishes occupied Floodwood (with less vegetation, mostly concentrated at channel edges). Furthermore, fish abundance and size structure appear to be related to vegetation itself, and not simply shallow habitat (e.g., 100% of Sterling and 97% of Floodwood bottom depths are within the euphotic zone). Vegetation may be of greater benefit to small-bodied than large-bodied fishes, because it provides zoobenthivores, such as the numerically dominant yellow perch and pumpkinseed, with diverse, abundant prey and protection from predation [1,26]. In embayments supporting a greater proportion of large-bodied fishes (e.g., Floodwood, Blind Sodus, and South Sandy), peak biomass was concentrated in fewer, but larger individuals. Neither embayment area nor piscivore biomass explained maximum biomass weight class, suggesting that both medium- and large-bodied fishes benefited from any deeper habitat associated with larger surface area.

Different distributions of biomass across fish size classes among embayments certainly could have implications for trophic cascades [38,10] and the susceptibility of some of these systems to shift from a macrophyte-to phytoplankton-dominated stable state [39]. For example, an ecosystem in which peak biomass occurs at larger size classes may be primarily structured by top-down effects. Such systems, such as Floodwood, may be less prone to undesirable eutrophication effects (e.g., algal blooms) due to piscivory of planktivorous and benthivorous fishes [10]. In contrast, a system such as Sterling, in which fish biomass is concentrated in smaller-bodied fishes may be more susceptible to eutrophication effects. In fact, zooplankton biovolume is low and phytoplankton biovolume is high in this embayment compared to the others (R. Doyle-Morin, Cornell University, personal communication). Certain fish communities may be an indication of top-down effects, while bottom-up (e.g., nutrient loading) control may play a greater role in other fish communities. In a study of yellow perch growth and size structure in four Lake Ontario embayments greater bottom-up control was observed in shallow embayments, whereas predation may play a more important role in deep, less vegetated embayments [40].

## Conclusion

Our study contributes to general understanding of how fish communities respond to physicochemical features both at the watershed and lake levels. Our findings suggest that fish communities are structured by factors operating at multiple spatial scales and on multiple community characteristics. Additionally, the importance of these factors appears to differ with hydrogeomorphology. Therefore, the relative impacts of natural variability and anthropogenic activity on fish communities in shallow, vegetated aquatic ecosystems are likely to differ somewhat from those in large, deep lakes. Influential factors of particular importance are those subject to human modification, such as percent vegetation and total phosphorus loading. For example, as water clarity has improved in the Great Lakes, macrophyte densities have increased to the extent that they are now considered a nuisance to nearshore activities and are being controlled through mechanical harvesting. Shoreline development and modification of connections between embayments and the main lake impact the quality of littoral habitat, integrity of adjacent wetland habitat, and water residence time. Additionally, these changes could alter fish movement into and out of the embayments as well as the quality of spawning habitat, two factors that were not considered in our study. Changing land use, such as the transformation of farmland to forested or urban land will continue to alter water discharge and nutrient and sediment loading. Identifying the actual mechanisms by which morphological and hydrological variables operate is challenging due to the degree to which many of these factors are correlated. However, developing a more explicit understanding of how these factors structure fish communities is important not only for coastal reclamation or restoration efforts along the Great Lakes coastline, but also for anticipating effects of future changes to inland, coastal, and offshore freshwater habitats and fish communities.

## Authors' contributions

KA contributed to the study's conception and design, conducted the research and data analysis, and wrote the manuscript. MB contributed to the study's conception and design, provided funding and resources for the research, and assisted with manuscript preparation.

## Supplementary Material

Additional file 1 **Morphometric, hydrologic, and land use characteristics of eight Lake Ontario embayments**. Annual total phosphorus load and water residence time were estimated from monthly data provided by X. Chen (unpublished data). Int = intermittent; Perm = permanent; Ssnl = seasonal; Trib = tributary; Wet = wetland. Click here for file

Additional file 2 **Embayment characteristics, 2001 – 2003**. Ranges (minimum and maximum) of values for mean station depth, percent vegetation, and secchi depth for each year. Species diversity (Simpson's diversity index) was calculated based on all individuals captured during July 2001–2003. Annual piscivore biomass (BPUE) was calculated for all piscivorous species. Quadratic equation r^2 ^values for maximum biomass weight class (h) estimates are shown in parentheses. Click here for file

Additional file 3 **2001 – 2003 catch per unit effort and biomass per unit effort means and standard errors for each focal species, all non-focal species combined, and all species combined**. CPUE (#·min^-1^) and BPUE (g·min^-1^) means and standard errors based on annual estimates for each embayment. Annual estimates were calculated as the sum of the number and biomass of fishes collected at all stations divided by the sum of the effort at all stations. Click here for file
