# Supplementary material for: Fish communities in coastal freshwater ecosystems: the role of the physical and chemical setting
Source: BMC Ecol. 2008 Dec 29;8:23. doi: 10.1186/1472-6785-8-23 (PMC2632619; doi:10.1186/1472-6785-8-23)
Supplement: Additional file 1 — Morphometric, hydrologic, and land use characteristics of eight Lake Ontario embayments. Annual total phosphorus load and water residence time were estimated from monthly data provided by X. Chen (unpublished data). Int = intermittent; Perm = permanent; Ssnl = seasonal; Trib = tributary; Wet = wetland. [file 1472-6785-8-23-S1.pdf]

Additional file 1.

	Western sites				Eastern sites			
	Blind Sodus	Little Sodus	Sterling	Juniper	South Sandy	North Sandy	South Colwell	Flood-wood
Watershed area (km^2)	35.2	8.56	210	0.60	8.26	210	1.38	672
% agricultural land use	36.7	17.4	41.1	40.3	29.2	24.2	19.4	37.2
Total phosphorus load (kg/yr)	596	31.9	4105	13.2	174	3043	10.2	17036
Embayment area (km2)	0.97	2.96	0.38	0.05	1.23	9.73	0.42	0.08
Maximum depth (m)	7.3	11	3.0	2.7	6.4	5.2	3.0	5.2
Mean depth (m)	3.60	4.75	0.85	1.22	2.06	2.36	1.26	0.73
Euphotic zone depth (m)	4.68	8.13	2.88	3.80	3.49	5.09	4.07	3.50
% euphotic zone area	0.49	0.57	1.00	1.00	0.54	0.99	1.00	0.97
Mean temperature (ºC) at depths ≤ 4 m	20.5	20.6	20.3	21.1	20.9	20.5	20.6	19.3
Water residence time (d)	47	56	1.3	62	148	35	90	0.1
Connection								
Lake Ontario	Ssnl	Perm	Perm	None	Ind	Perm	Int	Perm
Watershed	Trib	Int Trib	Trib	None	Wet/Trib	Trib	Wet	Trib
