# Supplementary material for: Fish communities in coastal freshwater ecosystems: the role of the physical and chemical setting
Source: BMC Ecol. 2008 Dec 29;8:23. doi: 10.1186/1472-6785-8-23 (PMC2632619; doi:10.1186/1472-6785-8-23)
Supplement: Additional file 2 — Embayment characteristics, 2001 – 2003. Ranges (minimum and maximum) of values for mean station depth, percent vegetation, and secchi depth for each year. Species diversity (Simpson's diversity index) was calculated based on all individuals captured during July 2001–2003. Annual piscivore biomass (BPUE) was calculated for all piscivorous species. Quadratic equation r2 values for maximum biomass weight class (h) estimates are shown in parentheses. [file 1472-6785-8-23-S2.pdf]

Additional file 2.

	Western sites				Eastern sites			
	Blind Sodus	Little Sodus	Sterling	Juniper	South Sandy	North Sandy	South Colwell	Flood-wood
CPUE (#·min^{-1})								
brown bullhead	0.06 (0.03)	0.12 (0.06)	0.20 (0.08)	0.04 (0.02)	0.04 (0.02)	0.15 (0.03)	0.07 (0.04)	0.11 (0.01)
bowfin	0.05 (0.02)	0.02 (0.01)	0.09 (0.04)	0.00	0.01 (0.005)	0.03 (0.02)	0.04 (0.01)	0.04 (0.02)
bluegill	0.11 (0.04)	0.07 (0.04)	0.15 (0.08)	0.00	0.10 (0.05)	0.08 (0.03)	0.39 (0.12)	0.54 (0.18)
golden shiner	0.10 (0.04)	0.04 (0.02)	0.19 (0.07)	0.19 (0.08)	0.05 (0.03)	0.04 (0.02)	0.04 (0.03)	0.02 (0.01)
largemouth bass	0.09 (0.01)	0.08 (0.02)	0.23 (0.04)	0.00	0.15 (0.14)	0.20 (0.14)	0.35 (0.14)	0.39 (0.05)
northern pike	0.04 (0.01)	0.003 (0.003)	0.06 (0.01)	0.00	0.03 (0.01)	0.01 (0.005)	0.02 (0.02)	0.04 (0.02)
pumpkinseed	0.19 (0.09)	0.26 (0.13)	1.9 (0.52)	0.13 (0.08)	0.20 (0.08)	0.22 (0.10)	0.36 (0.14)	0.45 (0.27)
walleye	0.00	0.00	0.00	0.00	0.07 (0.01)	0.00	0.00	0.00
yellow perch	1.1 (0.27)	0.96 (0.47)	1.6 (0.35)	0.18 (0.16)	0.45 (0.09)	0.87 (0.32)	1.2 (0.34)	0.32 (0.25)
non-focal species	0.21 (0.09)	0.08 (0.04)	0.21 (0.04)	0.08 (0.04)	0.13 (0.02)	0.15 (0.06)	0.32 (0.11)	0.43 (0.27)
all species	2.0 (0.46)	1.6 (0.75)	4.6 (0.59)	0.62 (0.34)	1.2 (0.12)	1.8 (0.45)	2.8 (0.18)	2.3 (0.56)
BPUE (g·min^{-1})								
brown bullhead	26 (14)	48 (18)	79 (36)	1.5 (0.92)	12 (3.7)	30 (8.9)	18 (15)	40 (5.7)
bowfin	103 (39)	24 (18)	142 (76)	0.00	16 (8.2)	64 (47)	54 (8.8)	56 (22)
bluegill	6.0 (2.6)	3.1 (1.8)	5.0 (0.66)	0.00	4.4 (2.4)	2.1 (1.2)	10 (2.7)	14 (5.5)
golden shiner	2.3 (1.8)	1.4 (0.80)	1.3 (0.41)	1.2 (0.34)	0.42 (0.30)	0.42 (0.08)	1.0 (0.78)	0.59 (0.33)
largemouth bass	14 (5.0)	24 (11)	7.9 (6.6)	0.00	0.54 (0.50)	27 (17)	15 (6.6)	26 (16)
northern pike	36 (12)	3.3 (3.3)	34 (4.8)	0.00	35 (11)	5.6 (3.0)	20 (20)	44 (19)
pumpkinseed	7.0 (2.9)	14 (7.2)	50 (18)	2.0 (1.7)	5.9 (1.2)	6.6 (4.1)	8.6 (1.1)	11 (7.1)
walleye	0.00	0.00	0.00	0.00	41 (14)	0.00	0.00	0.00
yellow perch	20 (2.3)	23 (12.4)	35 (1.3)	2.4 (2.2)	5.5 (2.6)	9.6 (2.7)	17 (5.5)	6.0 (4.7)
non-focal species	66 (59)	45 (24)	5.4 (3.4)	0.3 (0.20)	36 (30)	7.5 (5.8)	7.7 (4.0)	263 (126)
all species	280 (60)	187 (86)	361 (92)	7.3 (3.7)	156 (58)	154 (61)	152 (27)	461 (133)
