# Supplementary material for: Fish communities in coastal freshwater ecosystems: the role of the physical and chemical setting
Source: BMC Ecol. 2008 Dec 29;8:23. doi: 10.1186/1472-6785-8-23 (PMC2632619; doi:10.1186/1472-6785-8-23)
Supplement: Additional file 3 — 2001 – 2003 catch per unit effort and biomass per unit effort means and standard errors for each focal species, all non-focal species combined, and all species combined. CPUE (#·min-1) and BPUE (g·min-1) means and standard errors based on annual estimates for each embayment. Annual estimates were calculated as the sum of the number and biomass of fishes collected at all stations divided by the sum of the effort at all stations. [file 1472-6785-8-23-S3.pdf]

Additional file 3.

Embayment	Year	# stations	Mean depth (m)	Percent vegetation	Secchi depth (m)	Species diversity	Piscivore BPUE	*h*
Blind Sodus	2001	4	1.0 – 2.2	40 – 100	0.9 – 2.1	2.77	178.3	6.15 (0.52)
	2002	4	1.3 – 2.9	20 – 100	0.9 – 2.1		283.8	6.57 (0.47)
	2003	4	1.6 – 3.1	20 – 50	1.2 – 2.0		162.2	6.00 (0.46)
Little Sodus	2001	6	2.1 – 2.8	75 – 100	2.0 – 2.8	2.64	229.9	5.62 (0.28)
	2002	8	1.7 – 5.8	0 – 100	1.1 – 2.9		111.4	5.64 (0.48)
	2003	6	2.1 – 3.6	10 – 60	1.1 – 2.5		27.4	6.07 (0.24)
Sterling	2001	4	1.1 – 1.6	60 – 100	1.1	3.32	163.3	5.12 (0.17)
	2002	4	1.3 – 2.2	80 – 90	0.8 – 2.0		260.7	5.09 (0.39)
	2003	4	0.9 – 2.6	20 – 45	0.8 – 1.8		485.5	5.86 (0.45)
Juniper	2001	3	1.8 – 2.2	100	1.0	4.62	0	3.77 (0.27)
	2002	3	0.9 – 1.7	95 – 100	0.8 – 1.0		0	3.82 (0.48)
	2003	3	1.1 – 2.1	60 – 70	1.0 – 1.3		0	4.66 (0.45)
South Sandy	2001	4	1.2 – 2.5	–	0.9 – 1.0	5.08	58.90	6.74 (0.45)
	2002	5	1.3 – 3.1	0 – 50	1.0 – 1.2		173.7	7.25 (0.40)
	2003	4	1.3 – 3.5	0 – 10	0.7 – 0.9		107.0	5.72 (0.40)
North Sandy	2001	8	1.1 – 3.2	50 – 100	1.3 – 2.8	3.43	98.60	5.12 (0.35)
	2002	8	0.8 – 3.4	0 – 100	0.8 – 2.6		62.60	5.16 (0.52)
	2003	6	1.3 – 2.8	5 – 50	1.0 – 2.1		268.3	7.10 (0.28)
South Colwell	2001	4	1.5 – 2.0	100	1.8	4.05	82.90	5.15 (0.30)
	2002	4	1.6 – 1.8	50 – 100	1.1 – 1.2		167.6	5.64 (0.46)
	2003	5	1.1 – 2.1	40 – 50	0.9 – 1.9		143.9	4.87 (0.40)
Floodwood	2001	3	1.4 – 2.0	90 – 100	0.8	6.72	175.0	6.47 (0.24)
	2002	3	1.7 – 2.9	15 – 100	0.9 – 1.0		191.0	7.75 (0.71)
	2003	3	2.0 – 2.9	20 – 40	0.9 – 1.7		169.6	5.94 (0.5)
